# Forest riparian buffers reduce timber harvesting effects on stream temperature, but additional climate adaptation strategies are likely needed under future conditions

**DOI:** 10.2166/wcc.2020.031

**Published:** 2021-08-01

**Authors:** Hillary N. Yonce, Saumya Sarkar, Jonathan B. Butcher, Thomas E. Johnson, Susan H. Julius, Stephen D. LeDuc

**Affiliations:** Tetra Tech Inc., P.O. Box 14409, 1 Park Drive, Suite 200, Research Triangle Park, NC 27709, USA; Tetra Tech Inc., 12655 N. Central Expressway, Suite 305, Dallas, TX 75243, USA; Tetra Tech Inc., P.O. Box 14409, 1 Park Drive, Suite 200, Research Triangle Park, NC 27709, USA; US EPA Office of Research and Development, 1200 Pennsylvania Avenue, N.W., Washington, D.C. 20460, USA; US EPA Office of Research and Development, 1200 Pennsylvania Avenue, N.W., Washington, D.C. 20460, USA; US EPA Office of Research and Development, 109 TW Alexander Dr Research Triangle Park, NC 27709, USA

**Keywords:** climate sensitivity, forestry, QUAL2K, riparian buffer, SWAT, water temperature

## Abstract

Stream water temperature imposes metabolic constraints on the health of cold-water fish like salmonids. Timber harvesting can reduce stream shading leading to higher water temperatures, while also altering stream hydrology. In the Pacific Northwest, riparian buffer requirements are designed to mitigate these impacts; however, anticipated future changes in air temperature and precipitation could reduce the efficacy of these practices in protecting aquatic ecosystems. Using a combined modeling approach (Soil and Water Assessment Tool (SWAT), Shade, and QUAL2K), this study examines the effectiveness of riparian buffers in reducing impacts of timber harvest on stream water temperature in Lookout Creek, Oregon across a range of potential future climates. Simulations assess changes in riparian management alone, climate alone, and combined effects. Results suggest that maximum stream water temperatures during thermal stress events are projected to increase by 3.3–7.4 °C due to hydroclimatic change alone by the end of this century. Riparian management is effective in reducing stream temperature increases from timber harvesting alone but cannot fully counteract the additional effects of a warming climate. Overall, our findings suggest that the protection of sensitive aquatic species will likely require additional adaptation strategies, such as the protection or provisioning of cool water refugia, to enhance survival during maximum thermal stress events.

## INTRODUCTION

The effects of timber harvest on the thermal regime of streams have been observed globally from Oregon to Amazonia. Beginning in the 1950s, watershed studies of clearcut harvesting showed that removal of riparian vegetation resulted in dramatic increases in water temperature in the Oregon Coast Range (e.g., Brown & Krygier 1970). Reduction in baseflow, which can occur due to soil compaction during harvest, may also reduce the capacity to absorb increased thermal inputs. Preventing these adverse effects was a motivating factor in the development of riparian buffer management practices for forestry. Riparian buffers influence water temperature through shading that reduces direct insolation to streams, and secondary effects on microclimate (air temperature and wind) within the buffer are also important. Additional benefits of buffers include reducing peak storm runoff, maintaining stable baseflow, and potentially filtering out sediment, nutrients, and other pollutant loads from the surrounding area. Whether performance of riparian buffers under future conditions is sufficient to offset the effects of warming remains an open question, however.

There is growing concern about the potential effects of climate change on stream temperature. Stream temperatures are projected to increase in most North American rivers during the next century, resulting in increased stress on cold-water fish species such as salmonids. In the Pacific Northwest (PNW), air temperature increased from 1900 to 2000 by about 0.8 °C (Mote & Salathé 2010), mostly due to warming since 1976 (Isaak *et al.* 2018). Increases were larger for daily minimum temperature than for daily maximum temperature (Hamlet & Lettenmaier 2007). Future increases in air temperature in the PNW are projected to be 1.8–2.9 °C by the 2080s compared with climate 1970–1999 (Mote *et al.* 2014). Precipitation changes are less certain, but most models suggest wetter conditions in fall and winter, earlier snowmelt, and drier summers in the PNW.

Several studies have examined the potential impact of these changes on water temperatures in the PNW (e.g., Wu *et al.* 2012; Beechie *et al.* 2013; Dugdale *et al.* 2018). Results show that the thermal responses of individual streams depend on the complex interaction of hydrology, topography, and climate. Maximum water temperature in some PNW streams could be impacted more by reductions in summer streamflows, associated with drier and warmer conditions, than the direct impact of projected increases in air temperature (Cristea & Burges 2010; Wu *et al.* 2012). Streams that will shift from snow-dominant regimes to mixed or rain-dominant hydrology are also theorized to be more sensitive to increases in air temperature (Mote & Salathé 2010). Luce *et al.* (2014) showed that rain-dominated, low elevation watersheds were more sensitive to climate warming than streams draining steeper topography where flows are dominated by snowmelt in the PNW.

Reducing the risk of future water temperature increases requires understanding the interactions between hydroclimatic conditions and forest management practices. In this study, we examine the effectiveness of riparian buffers for reducing the impacts of timber harvest on stream temperature across a range of potential future changes in air temperature and precipitation. As our study system, we use Lookout Creek (LC), a mixed rain-and-snow catchment, representative of forested catchments on the western, windward side of the Oregon Cascades in a steep headwater catchment. Specifically, we address the following questions:

1. How will projected future changes in air temperature and precipitation affect water temperature in a small, forested PNW watershed?
2. How effective are forest riparian buffers in mitigating anticipated increases in water temperature?

## METHODS

In this study, we use an upland watershed model (Soil and Water Assessment Tool (SWAT)), a riparian vegetation shading model (Shade), and an instream hydraulic model (QUAL2K) to simulate streamflow and water temperature responses to a range of air temperature, precipitation, and forest management scenarios.

The SWAT is a lumped-parameter watershed model that simulates the watershed hydrologic response, including changes to summer baseflow, and provides the flow boundary conditions for the stream temperature model. The SWAT model was selected to represent boundary conditions to QUAL2K for the following reasons: (1) it is a widely accepted, tested, and often-used tool for predicting the impacts of climate and land management practices on hydrology at the watershed scale, (2) it incorporates explicit representation of plant growth needed to represent the effects of changes in temperature, precipitation, humidity, and ambient CO_2_ concentration on vegetation and hydrology, and (3) it can be successfully implemented with readily available GIS data. The impact of changes in CO_2_ concentrations in the SWAT model are manifested in the simulation of stomata control, which reduces water vapor losses by reducing leaf stomata conductance as CO_2_ concentrations rise (Easterling *et al.* 1992). The SWAT simulates water temperature, but in a simple manner that does not fully account for riparian shading and other aspects of the heat balance. The Shade model (developed by Washington Ecology; shade_ver40b04a06.xlsm; www.ecy.wa.gov/programs/eap/models.html) evaluates the effects of canopy and topographic shading on energy exchanges between the atmosphere and stream and is often paired with QUAL2K. QUAL2K is a steady-state stream water-quality model that provides a robust instream simulation of the heat balance during thermal stress events. QUAL2K is a one-dimensional model capable of representing a well-mixed channel with steady-state hydraulics, non-uniform steady flow, and diel heat budget and water-quality kinetics (Chapra *et al.* 2012; Version 2.12b1; http://www.qual2k.com/). The QUAL2K model is run for steady-state hydrologic conditions with diel variability in meteorology and shade.

### Study site

**Figure 1 |:**
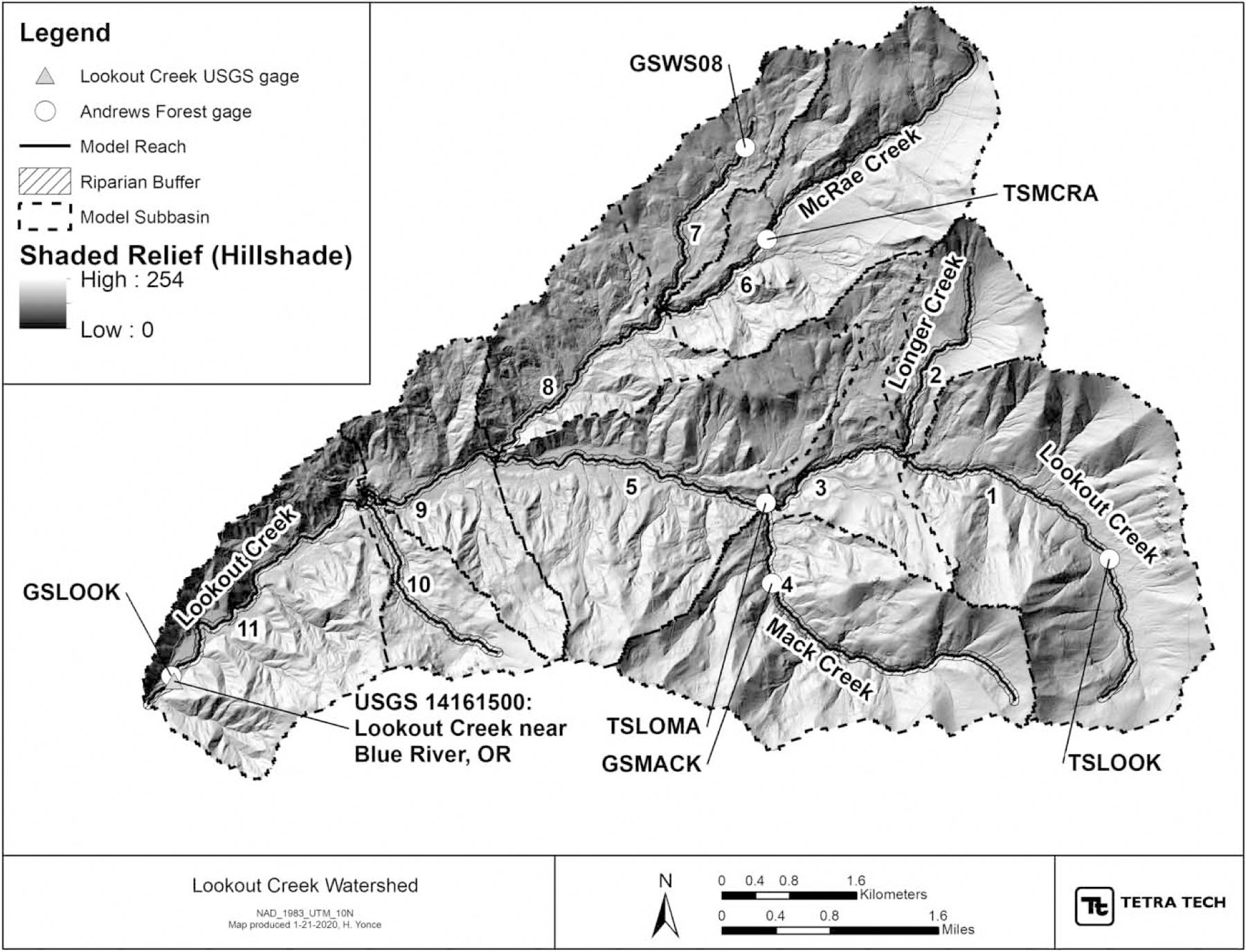
Subbasin boundaries, model reaches, riparian buffers, and monitoring sites in the LC watershed.

Model simulations were applied to LC, a fourth-order mountain headwater stream in the McKenzie 8-digit hydrologic unit code #17090004, tributary to the Willamette River in Oregon. The LC watershed lies completely within the United States Forest Service H. J. Andrews Experimental Forest (Andrews Forest) and is part of the Long-Term Ecological Research (LTER) network. The watershed occupies approximately 63.5 km^2^, with an elevation range from 420 to 1,630 m, and is almost entirely covered with old-growth conifer forest of Douglas fir (*Pseudotsuga menziesii*), western hemlock (*Tsuga heterophylla*), and western red cedar (*Thuja plicata*) (Figure 1). About 25% of the watershed was historically logged around 60–80 years ago; however, the riparian areas are almost exclusively densely vegetated old-growth conifers which provide ample shade. LC is generally steep (858-m change over 17.5 km; slope of 0.05) and is well-aerated, shaded, and cool with groundwater-fed baseflow. The climate of LC is wet and mild in winter and warm and dry in summer, typical of the Western Cascades, with winter snowpack historically present above 1,050 m. The stream supports salmonid populations of native cutthroat trout (*Oncorhynchus clarkii*) and non-native rainbow trout (*Oncorhynchus mykiss*).

Recently, there has been an observed increase in the number and duration of warm events per decade in the Cascades that may negatively affect aquatic biota. Historical data (1976–2015) throughout the PNW suggest that August air temperature in this region (zone H, Oregon Coastal) has increased by about 0.29 °C per decade, while August streamflow has decreased by nearly 4% (Isaak *et al.* 2017).

### Model development

The SWAT represents a watershed as subbasins subdivided into smaller hydrologic response units (HRUs). HRUs are unique combinations of land-use, soil, and slope classes. Land-use and soil property inputs for the LC watershed represented in the SWAT are based on the National Land Cover Database 2011 version (NLCD; 30-m resolution resampled to 10-m resolution) and the State Soil Geographic (STATSGO; polygon-based, converted to 10-m resolution raster). Slope is based on a 10-m resolution digital elevation model. Water yield is generated at the HRU level (10-m resolution) and aggregated by subbasin and transferred to the stream reach routing component of the SWAT. The stream reach routines simulate the transport and transformation of nutrients in the stream network. The SWAT application for the LC watershed consists of 111 HRUs (using two slope classes and zero thresholds for HRU land-use, soil, and slope definition) and 11 subbasins and reaches. The SWAT was configured to approximate observed streamflow and water quality in the LC watershed 2000–2015.

Meteorological forcing in the SWAT is based on gridded, daily weather data. Daily precipitation time series were developed for each subbasin using the PRISM (Parameter-elevation Relationships on Independent Slopes Model), a 4-km resolution daily gridded precipitation product (http://prism.oregonstate.edu). Meteorological data at a coarser scale of approximately 14-km resolution were retrieved from NLDAS2 (North American Land Data Assimilation System version 2) and processed to develop air temperature, relative humidity, solar radiation, and wind speed forcing series. Potential evapotranspiration (PET) was automatically computed by the SWAT during the simulation from these series using the Penman Monteith method. Both gridded datasets were area-weighted to the SWAT subbasins.

A QUAL2K model was developed for the LC mainstem and major tributaries to examine potential changes to water temperature during critical conditions for thermal stress and risk to biota. Each QUAL2K stream segment or ‘reach’ was subdivided into 0.1-km computational elements. Inputs for each QUAL2K reach include physical characteristics (e.g., bed slope and reach length), boundary conditions (diffuse flows to the stream network provided by the SWAT), meteorological data (hourly air temperature, dew point temperature, wind speed, and cloud cover), and shading (provided by the Shade model). Runoff to LC as simulated by the SWAT over a longer period of time is dominated by shallow subsurface pathways, and baseflow is supported solely by subsurface flow. Therefore, the watershed flow entering QUAL2K reaches is represented as nominal (0.01 m^3^/s) diffuse inflow at upstream headwaters. The key linkage between the SWAT and QUAL2K models for both baseline model development and scenario application is characterized by hydrological changes in baseflow.

QUAL2K reach length, width, slope, and elevation were developed from LIght Detection And Ranging (LiDAR) spatial datasets flown in August 2008 (data available from https://andrewsforest.oregonstate.edu/). Reach hydraulics were simulated using the rating curve method. Velocity (*U*, m/s) and depth (*H*, m) were generated as power functions of streamflow (*Q*, m^3^/s) produced by the SWAT and scaled to observed streamflow at the watershed outlet: *U* = *a* · *Q*^*b*^; and *H* = *A* · *Q*^*B*^. Exponents *b* and *B* were set to typical values of 0.43 and 0.45, respectively, as suggested in the QUAL2K documentation (Chapra *et al.* 2012). Coefficients *a* and *A* were approximated using observed channel geometry from Andrews Forest research (Anderson *et al.* 2005). The relationships between observed depth and drainage area were used along with field observations of channel width and velocity at the U.S. Geological Survey (USGS) gage #14161500 (LC near Blue River, OR) to estimate depth and velocity coefficients.

The term ‘critical conditions’ for the QUAL2K model application refers to forcing that causes the highest reasonably expected 7-day average daily maximum water temperature (MWAT). Daily water temperature data are available for LC at USGS gage #14161500 (2012–2017). The highest MWAT was observed for the week prior to 8 July 2015 (20.9 °C), so that was the date selected for QUAL2K calibration. The second highest annual 7-day average daily MWAT was observed for the week prior to 4 August 2014 (19.4 °C), so that date was selected for model corroboration. A second corroboration application was constructed for 28 July 2013, the day which exhibited the highest 7-day average of the maximum air temperature (32.7 °C) at the Andrews Forest primary weather station (PRIMET).

Air temperature datasets for QUAL2K were constructed by reach using observed daily minimum and maximum temperatures from Andrews Forest meteorological stations averaged over the preceding week and interpolated to hourly values. Dew point temperatures were calculated using the hourly air temperature inputs and the average daily relative humidity observed across the watershed for the week prior to the simulation date. Hourly wind speeds were based on average observed summer conditions across the watershed, disaggregated to hourly inputs. Hourly cloud cover during daylight hours was based on observed solar radiation at the primary meteorological gage in the Andrews Forest and estimated cloudless sky radiation. Night cloud cover estimates were based on weather observations at Mahlon Sweet Airport in Eugene, OR (GHCND: USW00024221), which is the closest first-order station in the region.

Heat content from groundwater inflows and small, groundwater-driven tributaries has an important effect on mainstem water temperature in LC (Tague *et al.* 2008). To estimate these inputs for QUAL2K, we used data from five continuous water temperature monitoring sites on small streams in the watershed that were not included in the explicitly modeled stream network. These were used to develop a regression against elevation for the temperature of discharging groundwater. The combined impact of groundwater to the system was input to QUAL2K as steady diffuse inflow along the length of each model reach.

Stream shading associated with different riparian management scenarios was estimated using the Shade model. We used TTools, a GIS extension for sampling geospatial data on channel morphology, land cover, and topography, to assemble high-resolution inputs for the Shade model (available through Washington Ecology; www.ecy.wa.gov/programs/eap/models.html). TTools was used to sample August 2008 LiDAR-derived topography and vegetation height rasters, measure topographic angles, and stream gradients. Longitudinal GIS sampling output from TTools was used to tabulate hourly effective stream shading to the water due to topography and vegetation in the Shade model. The Shade model was run for the calibration model date (8 July 2015), and hourly shade estimates were averaged by model reach. The average daily shade from topography and vegetation for the 8 July 2015 calibration model was 69.2%. Average shade for the mainstem is lower than the tributaries (58.9% compared with 80.9%), which are narrower channels with a greater frequency of north–south orientation.

We conducted sensitivity analyses to test the relative impact of major model inputs on temperature predictions from the QUAL2K models. Results are summarized per QUAL2K output as either diel output at a single point instream or longitudinally from headwaters to the outlet. Of the parameters tested, simulated water temperature was less sensitive to changes in cloud cover and wind speed, and more sensitive to changes in air temperature, groundwater temperature, and shade.

### Model calibration approach

The calibration of the QUAL2K model focused on minimizing mean error and mean absolute error between simulated and observed water temperatures during warm summer conditions. Water temperature data were available for comparison at USGS site 14161500, and Andrews Forest monitoring gages on simulated reaches: GSLOOK, GSMACK, GSW08, TSLOMA, TSLOOK, and TSMCRA (Figure 1). We used the sensitivity analysis to identify key input parameters for calibration. The performance of the model was corroborated through application to two additional dates with high air and water temperatures.

The SWAT application was calibrated against observed continuous streamflow at the USGS gage. Moriasi *et al.* (2007) provide guidelines for the evaluation of SWAT applications based on Nash–Sutcliffe Efficiency (NSE) and percent bias (PBIAS) at a monthly time step. We also examined fit of daily time series and checked for seasonal biases as part of the calibration exercise.

### Scenario development

#### Climate scenarios

Scenarios representing a range of potential future climatic conditions for LC were developed based on the statistically downscaled global climate model (GCM) output from the Multivariate Constructed Analogs (MACA) version 2 archive (http://maca.northwestknowledge.net/). The constructed analog approach uses a library of historical observations to scale from monthly to daily time step, ensuring a reasonable representation of the temporal structure of local rainfall (Abatzoglou & Brown 2012). The MACA dataset also provides simultaneous downscaling of precipitation, temperature maxima and minima, humidity, wind, and radiation, helping to ensure physical consistency in the outputs and allowing energy–balance estimation of PET. We selected a set of five downscaled GCM projections (Table 1) to approximate a plausible range of potential future changes. Specifically, we selected GCMs that approximate the median, 10th, and 90th percentiles of changes in annual average precipitation and air temperature assuming relatively high greenhouse gas projections (Coupled Model Intercomparison Project Phase 5 [CMIP5] Representative Concentration Pathway [RCP] 8.5). We focus on GCM output for late-century conditions (2071–2100) to provide a wider range of possible conditions and because achieving maximum riparian vegetation is a process that takes decades. Projected results for intermediate time horizons will fall along a trajectory between the historic and end-of-century scenarios, although the form of that trajectory is not known.

Daily gridded MACA precipitation, maximum and minimum air temperature, solar radiation, wind speed, and relative humidity data were area-weighted and aggregated by SWAT subbasins for the LC watershed for the hindcast (1950–2005) and forecast (2069–2099) periods. While we simulate the hindcast period (1950–2005), we report changes in water yield for the forecast period relative to the last 30 years (1976–2005).

Atmospheric CO_2_ concentration also has direct effects on plant growth and physiology, which has a potentially significant impact on evapotranspiration (ET). Plants decrease stomatal conductance to mitigate transpiration water loss as atmospheric CO_2_ concentrations rise. Ambient CO_2_ concentrations at the midpoint of each simulation period were specified from the IPCC CMIP5 estimates (historic [1978] – 335 ppm; mid-century [2045] – 513 ppm; late century [2085] – 801 ppm). The SWAT then reduces water vapor losses by reducing stomata conductance as CO_2_ concentrations rise (Easterling *et al.* 1992). Plant radiation use efficiency also changes in response to increased CO_2_.

The QUAL2K model inputs are steady-state representations of critical periods for which high water temperatures are expected, and meteorological inputs were developed from the same MACA datasets used with the SWAT. Air temperature inputs for QUAL2K scenarios represent the absolute increase in 7-day average daily maximum air temperatures from hindcast (1976–2005) to forecast (2070–2099) periods as applied to the calibration model. Dew point temperature inputs were calculated as a function of air temperatures and average relative humidity for the simulation date in the forecast period. Scenario boundary conditions for flows in QUAL2K were developed using model output from the SWAT, with the percent change in lowest annual 7-day average flow between hindcast and forecast periods applied to the existing condition flow rates. The reach-averaged change in baseflow for each climate scenario as simulated by the SWAT ranged from −0.7 to 22.4% across all GCMs. QUAL2K inputs for boundary condition (primarily diffuse inflow) water temperatures were estimated as the difference between average daily air temperatures over the hindcast and forecast periods, as shallow groundwater temperatures tend to reflect average annual air temperatures.

#### Forest management/shade scenarios

The buffer width needed to maintain shading varies with aspect, but almost all the shade effect takes place within about one tree height of the stream edge (e.g., within about 50–60 m for old-growth conifers in the PNW) (Moore *et al.* 2005). Groom *et al.* (2011) reported that 68–75% of post-harvest shade in western Oregon streams were accounted for by basal area within 30 m of the stream and that 30 m riparian buffers minimized post-harvest temperature increases.

For modeling purposes, the riparian buffer zone width was defined as 54 m on either side of all stream channels to incorporate the approximate distance at which effects on stream shade approach zero (Groom *et al.* 2011). Management options in the riparian buffer are explicitly represented in Shade/QUAL2K, ranging from clearcut to restoration of system potential vegetation (SPV), and including two partial harvest options within the buffer, as summarized in Table 2. Shade reported in the table is the reduction of daily insolation caused by topography and vegetation, before accounting for reflection from the water surface.

The SWAT does not provide a spatially explicit way to represent changes within the riparian buffer; therefore, scenarios for forest clearcutting and thinning were applied to all forest HRUs. The results are then extrapolated to the fraction of forest area lying within the buffer zone, which captures the basic upstream impact on hydrology for boundary condition inputs, while Shade and QUAL2K capture the key local effects on the stream. We evaluated four clearcutting scenarios; simulated on 5, 10, 20, and 50% of all forested HRUs. To simulate the immediate after-effects of clearcutting, we set the initial biomass and leaf area index (LAI) for affected HRUs to zero. We also increased the initial curve number (CN2) to be representative of barren or no vegetation conditions consistent with previous SWAT studies. For thinning, we also evaluated application to 5, 10, 20, and 50% of all forested HRUs. Forest thinning was represented by reducing the initial biomass and LAI limit of forested HRUs to half of their initial values.

## RESULTS AND DISCUSSION

### Results: model performance

**Figure 2 |:**
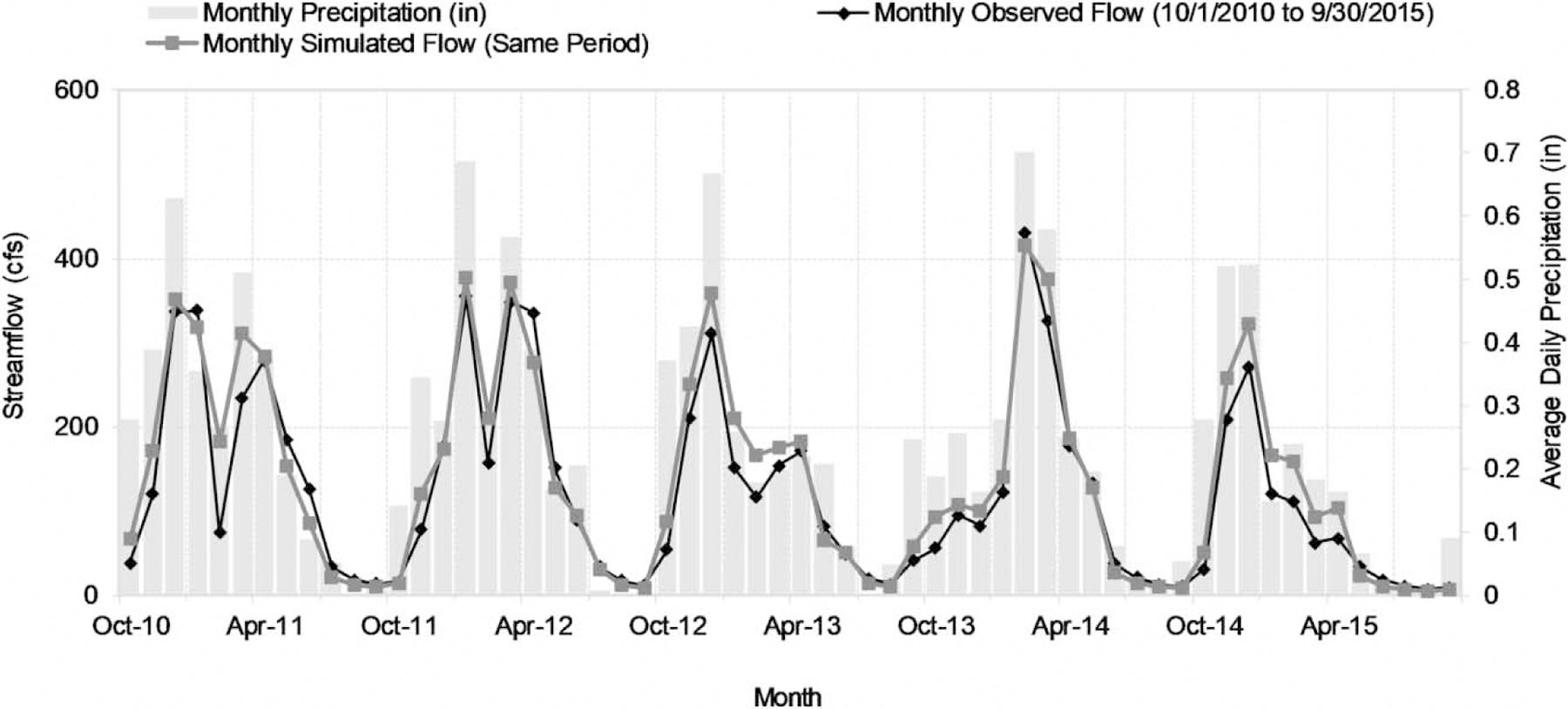
SWAT-simulated and -observed streamflow at USGS gage #14161500.

SWAT-simulated daily flow time series show good agreement with observations at the LC outlet (USGS #14161500; Figure 2). Daily NSEs for the calibration and validation periods (water years 2011–2015 and 2006–2010) are 0.749 and 0.674, respectively. Monthly NSE and PBIAS for the calibration and validation periods are 0.918 and 11.3%, and 0.826 and 18.5%, respectively. Based on the NSE alone, the model performance is rated as ‘very good’ based on the criteria of Moriasi *et al.* (2007), although the relatively high PBIAS puts the model performance in the ‘good’ to ‘satisfactory’ range. Comparison of flow duration curves shows that the model tends to overestimate midrange stream flows in the winter but provides an accurate representation of late summer baseflow conditions when water temperature maxima occur.

**Figure 3 |:**
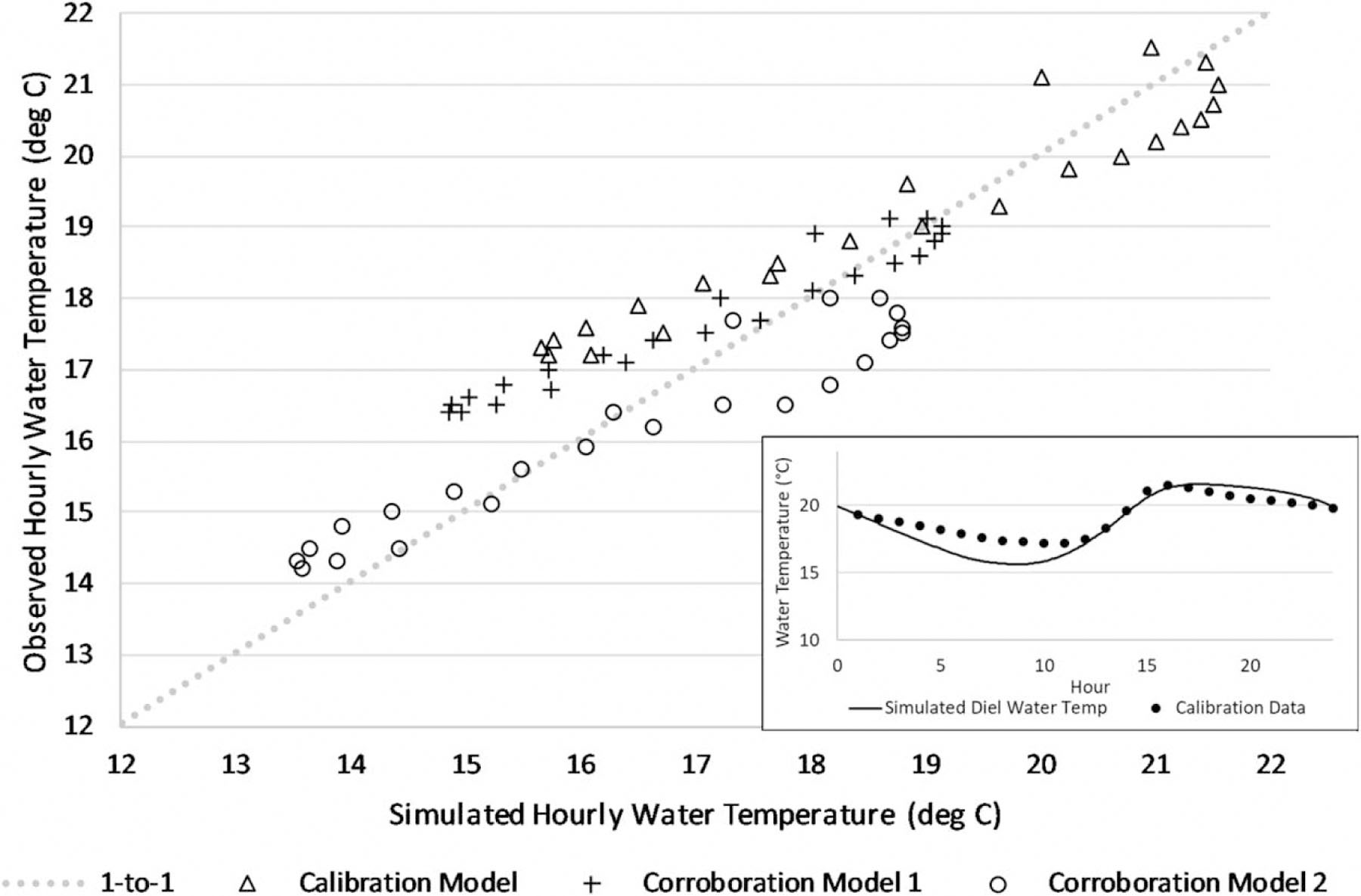
QUAL2K-simulated and -observed hourly water temperature time series at Andrews Forest gage GSLOOK.

QUAL2K-simulated water temperatures for both the calibration and corroboration models provide good matches to observed data at the watershed outlet, especially for the daily maximum (Table 3). The simulated daily temperature cycles were also reasonably accurate (Figure 3).

### Results: stream response

**Figure 4 |:**
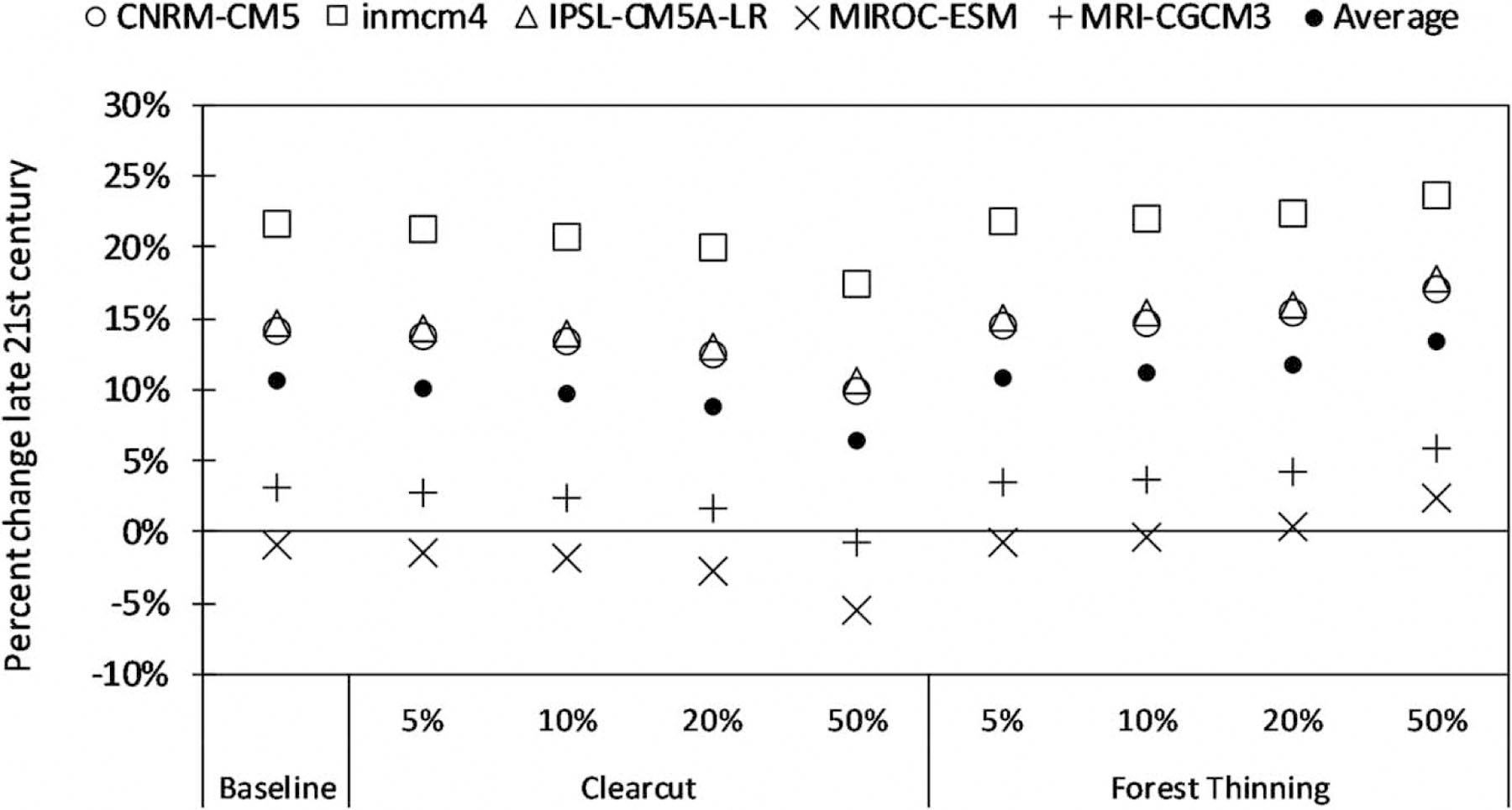
Change in the projected future annual water yield relative to historic conditions.

Simulated total water yield (Figure 4) increases in four out of five future scenarios by late century (2071–2100), with additional increases in yield associated with forest thinning, which reduces ET. SWAT simulations for clearcut scenarios represent changes in the first year after harvest. Under the clearcut scenario, the SWAT simulates a decrease in groundwater discharge and an increase in ET associated with rapid regrowth. The net result is a slightly smaller increase in water yield compared with simulations of current forest cover under future climate.

**Figure 5 |:**
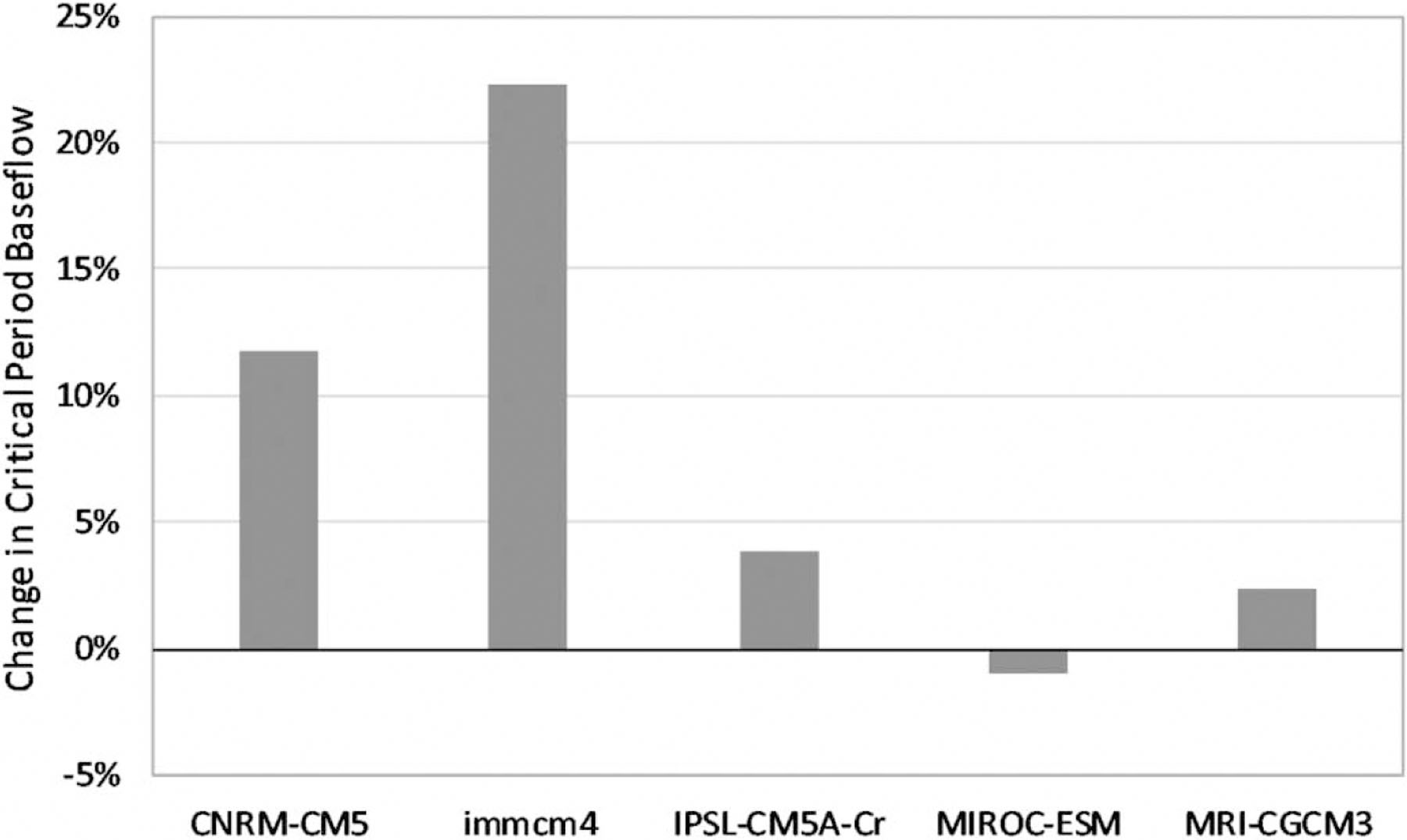
Late-century changes in baseflow for critical thermal stress events by GCM with the existing shade conditions.

The calibrated model run for the event of 8 July 2015 is referred to hereafter as ‘historic climate’ with the ‘existing shade’ for which scenario responses will be compared to as relative change. SWAT hydrologic simulations under future climate show precipitation increasing more than ET in four of five late-century scenarios (all but scenario MIROC-ESM), leading to an overall increase in water yield and critical period baseflow volume under existing shade conditions (Figure 5). This result is due to change in the net balance between input precipitation and outputs such as ET.

**Figure 6 |:**
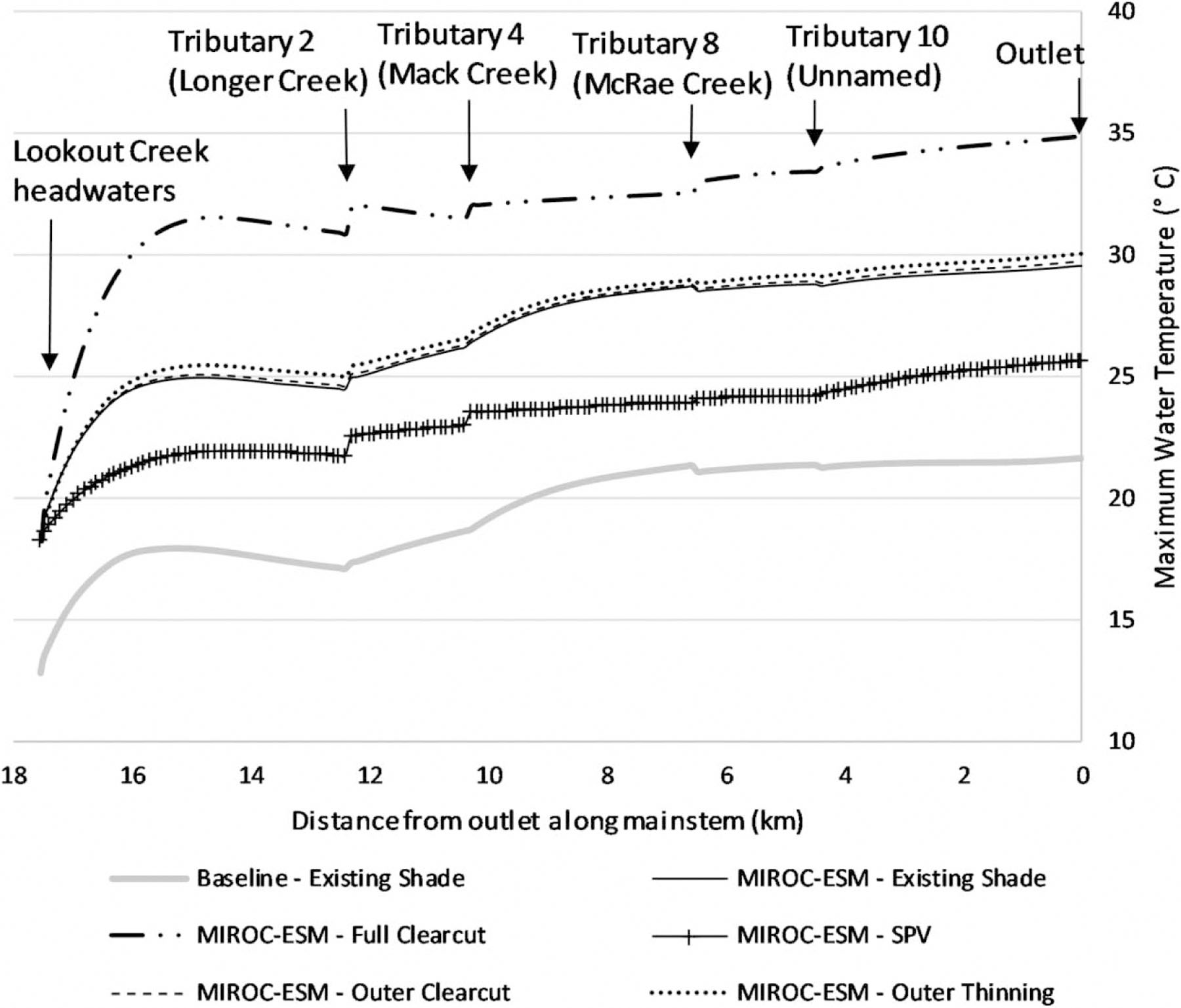
Longitudinal temperature changes along the LC mainstem for late-century (2071–2100) climate, MIROC-ESM GCM compared with historic conditions with the existing shade. *Note that cold headwater temperatures are depicted at the far left of the graphic, and so increases in water temperature shown as LC creek flows downstream are due to the impact of solar radiation, shade, and tributary inflows*.

Forest management and shade effects on water temperature were evaluated longitudinally along LC per QUAL2K model output from headwaters to outlet. Under historic climate, longitudinal simulation results for the MWAT show that clearcutting can increase temperatures throughout the length of LC, while SPV can reduce temperatures. A similar pattern is seen under late-century conditions, but the baseline is shifted upward due to increased thermal inputs (Figure 6).

Longitudinal results were spatially averaged across all model reaches and are summarized in Table 4. Under historic climate, clearcutting leads to large increases in water temperatures throughout LC. The most aggressive management response, the SPV scenario, can reduce temperatures, but not to baseline conditions under most late-century climate scenarios. A similar pattern is seen under late-century conditions; however, overall temperatures are increased due to warmer air temperatures. QUAL2K simulations suggest that in the absence of modified management practices (i.e., maintaining existing riparian conditions), MWATs are anticipated to rise from 17 to 38% during extreme thermal events. The results for each forest management practice show that clearcutting increases MWATs by nearly 30%, while SPV decreases MWATs by nearly 20% under historic climate. Riparian buffer outer clearcut and outer thinning scenarios result in only small increases in MWATs for LC relative to the existing shade because the intact portion of the buffer adjacent to the narrow stream channel is sufficient to fully shade the water surface.

**Figure 7 |:**
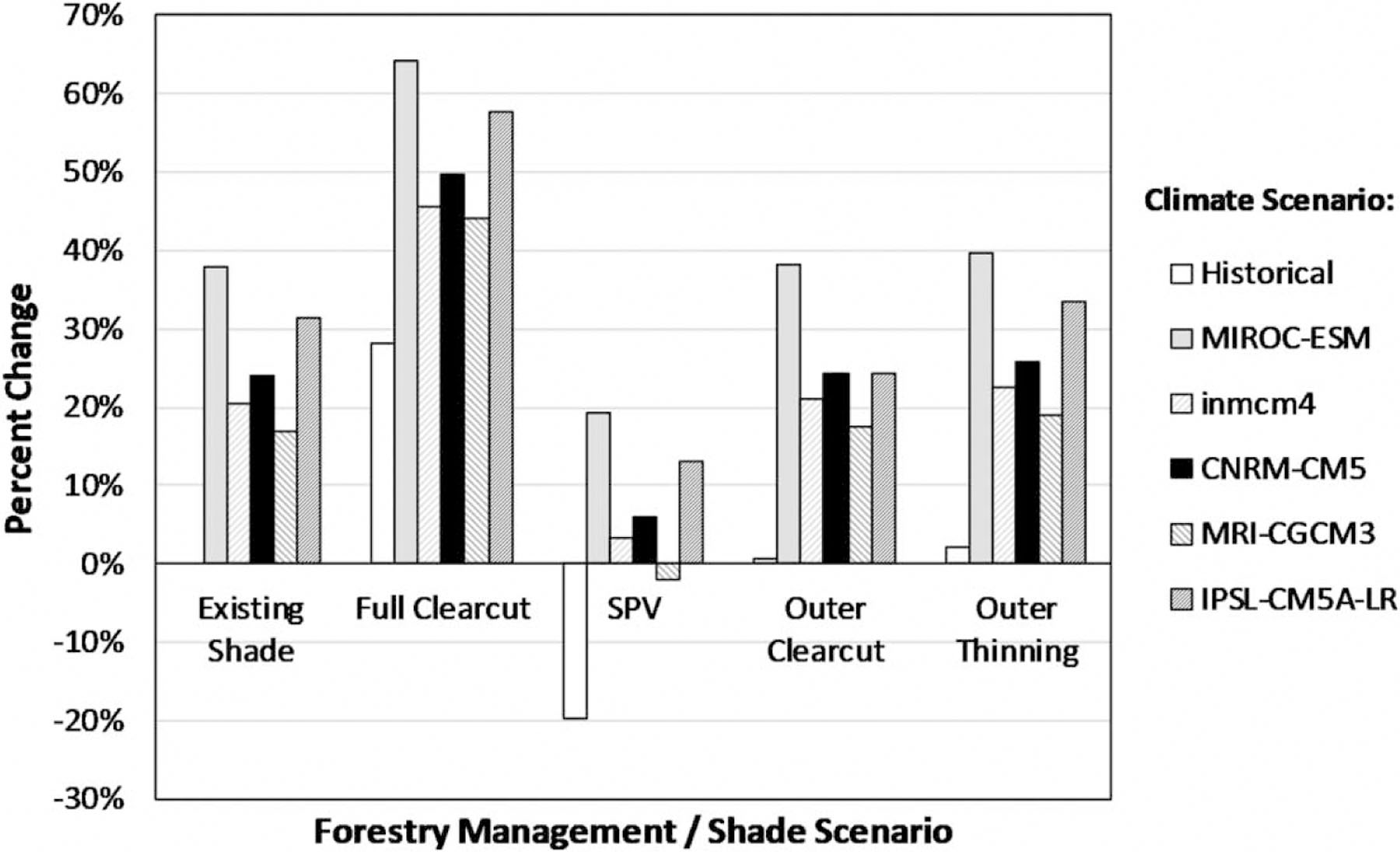
Late-century percent change in daily MWAT during critical thermal stress conditions relative to historic climate and the existing shade.

All future climate scenarios suggest future increases in MWAT, amplified by clearcutting, or mitigated by SPV. Among the GCMs considered in this study, MIROC-ESM has the largest increase in future air temperature (~7.4 °C warmer than historic) and the lowest projected future baseflow volume. MRI-CGCM3 has the smallest increase in future air temperatures (~3.3 °C warmer than historic). The simulated water temperatures under future conditions closely follow the trends in air temperature. Figure 7 summarizes the corresponding changes in the MWAT during critical thermal stress conditions.

Note that while simulations indicate that SPV provides a consistent benefit relative to other scenarios, it is not sufficient to mitigate projected increases in the MWAT by the end of century, as MWAT is still projected to increase in four out of five climate scenarios, while average water temperatures increase in all five.

Linked to the stream temperature response, QUAL2K also simulated decreases in dissolved oxygen (DO) concentrations which are integral drivers of stream ecological health. The decline in DO is mostly due to increases in water temperature, which reduces the attainable DO saturation concentration. DO concentrations were projected to decrease by nearly 10% or 1 mg/l during summer critical events.

## DISCUSSION

This study evaluates the effectiveness of riparian buffers for reducing the impacts of timber harvest on stream temperature in LC, a forested catchment in the Oregon Western Cascades, across a range of potential future changes in air temperature and precipitation. We first consider how potential future changes in air temperature and precipitation could affect water temperature. Simulations in this study suggest precipitation increasing more than ET, leading to increases in annual average water yield for the LC watershed. Shifts in the snow regime do not appear to be a major factor for future conditions in LC, which is already a mixed snow and rain system and is expected to remain so by late century. Despite increasing air temperatures and reduced winter snowpack, no significant decline in summer baseflow is projected. In contrast, a study of the entire Columbia River basin by Hamlet *et al.* (2013) suggested that summer low flows will decline in most streams west of the Cascades due to increased ET. The difference could be due, in part, to reduced ET, offsetting the effects of increasing temperatures, in our SWAT simulations resulting from representation of vegetative response to increased atmospheric CO_2_ concentrations.

In snow-dominant regimes, much of the winter precipitation is stored in snowpack, which melts in spring and early summer, resulting in peak flows in early spring, maintenance of baseflow, and a continued supply of cold melt water through summer (Hamlet & Lettenmaier 2007). Where air temperature increases, snow-dominant systems shift to mixed or rainfall-dominant regimes, significant reductions in summer flows and cool groundwater discharge are likely, potentially exacerbating the effects of increased air temperatures (Mote & Salathé 2010). Streams with marginal snow-dominant hydrology (Butcher *et al.* 2016) may be particularly sensitive due to a shift from winter snow to rain. Luce *et al.* (2014) and Isaak *et al.* (2016a) both noted that observed rates of water temperature change relative to air temperature change are typically lower in headwater streams because of short residence times and strong local temperature gradients associated with topography.

Despite minimal change in summer baseflow, our results suggest that stream temperatures will increase under future conditions. The QUAL2K simulation results suggest that MWATs during critical thermal stress events in LC will increase 17–38% (3.3–7.4 °C) under an existing riparian buffer condition by late century. The projected temperature increases are greater than the 2–3 °C increases in average summer temperatures suggested by Wu *et al.* (2012) for the end of century in this region, and the summer temperature increase of about 2 °C in LC suggested by Isaak *et al.* (2016b). Simulations reported by Wu *et al.* and Isaak *et al.* are based on the CMIP3 A1B scenario, which projects substantially lower air temperature increases by the end of the 21st century than the CMIP5 RCP 8.5 scenario used in this study. We also expect that extreme thermal events (MWATs) may increase by more than the summer average, and it is these extreme events that will impose metabolic constraints on fish species survival.

A second question we address is how effective forest management strategies using riparian buffers are to mitigate anticipated increases in water temperature in forest streams. We consider five forest management options simulated in QUAL2K representing a range of disturbances in the riparian buffer, from clearcutting (full loss of vegetative shade) to SPV, the expected condition of maximal shade absent anthropogenic disturbance. Resulting MWAT varies in accordance with this gradient in shade provided by riparian vegetation. The lowest MWAT is associated with SPV. It is important to note that riparian areas in most forests are not currently at SPV because of past activities.

QUAL2K simulation results suggest that MWATs under summer critical conditions are likely to increase under most late-century scenarios, regardless of riparian management practices. Restoration of full SPV in the riparian buffer can reduce temperatures relative to current vegetation under future climate but does not provide sufficient mitigation to prevent substantial temperature increases during critical thermal stress events relative to the historic temperature regime. Similar results have been suggested elsewhere in the PNW (e.g., Cristea & Burges 2010; Mantua *et al.* 2010; Butcher *et al.* 2016),

Simulations suggest that all riparian buffer strategies are effective in offsetting water temperature increases associated with harvest. Notably, the riparian management scenarios designed to allow harvest while protecting streamside shade resulted in an increase in MWATs relative to existing vegetation conditions, but only by a small amount (a maximum predicted increase of 0.4 °C). However, in most cases, riparian buffers cannot fully compensate/offset the possible increase in thermal stress events due to projected future increases in air temperature. This finding is consistent with findings from a modeling study of the South Fork Nooksack River (WA), which predicted that the risk of higher water temperatures will accelerate over time and is likely to overwhelm the mitigation provided by increased shade from riparian buffers (Butcher *et al.* 2016).

Our findings suggest that additional strategies beyond SPV will be necessary to meet temperature criteria designed to protect cold-water species. Protecting and restoring local cold-water refugia – such as log-jam pools, small tributaries, side channels, and backwaters fed by groundwater seeps or hyporheic upwelling – may help to mitigate the effects of warming temperatures on aquatic life during high-temperature events, as fish can thermoregulate by moving to such microhabitats (Beechie *et al.* 2013). The effectiveness of these strategies under future conditions, in combination with riparian buffer restoration, should be further examined.

As in all studies, there are several limitations which should be noted. First, effective riparian buffer widths are likely to vary depending upon the physical setting. LC has a mountain valley hydrologic landscape, with relatively steep hillslopes and very little flat riparian area. These hillslopes provide significant shading to the stream regardless of forest management. Thus, buffer widths likely be relatively smaller in mountain valley hydrologic landscapes than other less dissected landscapes. To achieve the same temperature-moderating effects, wider buffers may be needed in hydrologic landscapes with wider, flatter riparian areas and less significant topographical shading.

Second, impacts in the QUAL2K model from various forestry management techniques were largely limited to impacts on localized hydrology and baseflow. The realized impact of changing climate patterns on vegetation and the potential interactions between climate and management scenarios would require further analysis and different modeling applications. Mature SPV within the riparian corridor will be more resilient to changes in climate than less well-established communities; however, changing hydrologic and thermal regimes may also impact which species thrive relative to changes in PET in response to changes in CO_2_. Higher CO_2_ concentrations may increase mortality rates in case of drought due to an enhanced dependence on soil moisture as watershed hydrology changes. Watershed elevation also plays a role in the variable forest response to changing temperature and CO_2_ concentrations. The ecosystem response to changing CO_2_ is also dependent on tree species, age, stand density, stand health in response to pests, management or natural disturbance, seasonality, and nutrient availability which are not only difficult to simulate and predict, but have combined impacts as well on both forest response and management options and opportunities (Kallarackal & Roby 2012). For example, the increased likelihood of wildfire due to changing climate may be reduced by thinning operations. These interdependencies stress the need for whole forest ecosystem modeling to further analyze the effects and interactions between climate, vegetation, and management.

Finally, it should be noted that the results of this study are conditional on the methods, models, and scenarios used. The QUAL2K model implemented here may omit some important physical factors that could ameliorate water temperatures such as increased hyporheic flow or discharges of deeper, cooler groundwater. Conversely, increased tree mortality or changes in species composition under a future climate might yield incorrect assumptions about the amount of shading that can be achieved with SPV, although strategies are available to increase the climate resilience of riparian buffers.

## CONCLUSIONS

This study evaluates the effectiveness of riparian buffers for reducing impacts of timber harvest on stream temperature in LC, a forested catchment in the Oregon Western Cascades, across a range of potential future changes in air temperature and precipitation. Results suggest that riparian shade can play an important role in mitigating extreme water temperature events and can remain an effective practice for counteracting the effects of timber harvesting; however, anticipated temperature increases by late century will likely be greater than can be addressed through maintaining – or even increasing – riparian shade alone. While results presented are specific to LC in Oregon, they illustrate the risk of future temperature increases in other mid-altitude PNW streams. Although high-temperature events will likely increase even with attainment of SPV in riparian areas, achieving SPV will produce significantly better outcomes than scenarios under which riparian buffers are only partially protected. In addition to robust riparian buffers, other climate adaptation strategies, such as providing and/or protecting cold-water refugia, are likely needed to protect cold-water species from higher stream temperature events in the future.

## Figures and Tables

**Table 1 |:** GCMs selected for LC future climate simulations (CMIP5, RCP 8.5)

Future climate condition (relative increases)	GCM
10th percentile precipitation, 10th percentile air temperature	MRI-GCM3
90th percentile precipitation, 10th percentile air temperature	inmcm4
90th percentile precipitation, 90th percentile air temperature	IPSL-CM5A-LR
10th percentile precipitation, 90th percentile air temperature	MIROC-ESM
50th percentile precipitation, 50th percentile air temperature	CNRM-CM5

**Table 2 |:** Riparian forestry buffer management and shade simulation scenarios

Forest riparian buffer shade scenario	Shade results and QUAL2K components
Existing shade	LiDAR/TTools/Shade analysis of existing vegetation conditions. Average daily shade 69% (59% for mainstem and 68–87% for tributaries).
SPV	100-year SPV in riparian buffer with 85% density, tree height 50 m, overhang 3 m. Average daily shade 95% (93% for mainstem and 95–97% for tributaries).
Full clearcut	Fully clearcut, stream shading provided only by topography. Average daily shade 13% (9% for mainstem and 12–21% for tributaries).
Outer clearcut	Inner 24 m maintained at the existing shade, outer riparian areas clearcut. Average daily shade 68% (57% for mainstem and 67–87% for tributaries).
Outer thinning	Riparian buffer simulated with inner 6 m maintained at the existing shade, then 18 m of forest thinning (existing vegetation with density decreased by 25% and clearcut the rest of the outer buffer). Average daily shade 65% (54% for mainstem and 64–82% for tributaries).

**Table 3 |:** QUAL2K-simulated and -observed hourly water temperature statistics at Andrews Forest gage GSLOOK

Simulation date	Model type	Simulated mean (°C)	Observed mean (°C)	Simulated maximum (°C)	Observed maximum (°C)	Mean error	Mean absolute error
8 July 2015	Calibration	18.79	19.18	21.56	21.50	−0.39	0.86
4 August 2014	Corroboration	17.09	17.71	19.17	19.10	−0.62	0.74
28 July 2013	Corroboration	16.37	16.13	18.81	18.00	0.24	0.68

**Table 4 |:** Future maximum (spatially averaged) water temperature (°C and % change relative to historic climate and existing forest cover) for critical thermal stress conditions

Climate scenario	Forestry management/shade scenario				
	Existing shade	Full clearcut	SPV	Outer clearcut	Outer thinning
Historic	19.5	25.0 (28.1%)	15.7 ( − 19.7%)	19.6 (0.6%)	19.9 (2.2%)
MIROC-ESM	26.9 (37.8%)	32.0 (64.1%)	23.3 (19.3%)	27.0 (38.3%)	27.3 (39.8%)
inmcm4	23.5 (20.6%)	28.4 (45.5%)	20.2 (3.3%)	23.6 (21.0%)	23.9 (22.5%)
CNRM-CM5	24.2 (23.9%)	29.2 (49.7%)	20.7 (6.0%)	24.3 (24.4%)	24.6 (25.9%)
MRI-CGCM3	22.8 (17.0%)	28.1 (44.1%)	19.1 ( − 1.9%)	22.9 (17.5%)	23.2 (19.1%)
IPSL-CM5A-LR	25.7 (31.5%)	30.8 (57.7%)	22.1 (13.2%)	24.3 (24.4%)	26.1 (33.5%)

## Data Availability

All relevant data are included in the paper or its Supplementary Information.

## HIGHLIGHTS

- A range of future climate conditions can negatively impact cold-water fish species during extreme thermal events.
- Riparian forest management can help play a role to mitigate impacts of future climate conditions.
- Cold-water species may be unable to thrive in pristine traditional ecosystems due to climate impacts which cannot be mitigated during extreme thermal events.
- Mature forests are unlikely to shelter pristine freshwater streams from impacts of climate change.
- The importance of cold-water refugia and alternative mitigation strategies are likely to become increasingly important by the end of the century to combat climate change.
